# Matrix-assisted laser desorption ionization-time of flight mass spectrometry (MALDI-TOF) score algorithm for identification of *Gordonia* species

**DOI:** 10.1186/s13568-018-0650-z

**Published:** 2018-07-23

**Authors:** Maria Ercibengoa Arana, Marta Alonso, Pedro Idigoras, Diego Vicente, Jose María Marimón

**Affiliations:** 1 CIBER de Enfermedades Respiratorias-CIBERES, Madrid, Spain; 2 Preventive Medicine and Public Health Department, University of Basque Country, Donostia, Spain; 3 Microbiology Department, Donostia University Hospital-Biodonostia Health Institute, Paseo Dr. Beguiristain s/n, 20014 Donostia-San Sebastian, Spain

**Keywords:** *Gordonia* species identification, MALDI-TOF MS, Molecular technology, Discriminatory peaks

## Abstract

*Gordonia* species differentiation is a tedious task. Herein, *Gordonia* identification was performed according to the standard Bruker score system and a recently proposed score for Gram positive rods identification (≥ 1.5 genus level and ≥ 1.7 species level). New scores significantly improved the identification at genus and species level.

## Introduction

The bacterial genus Gordonia currently includes 39 different species (Sowani et al. 2017). This genus is particularly known for its biotechnological applications and it is also notable for being an opportunistic human pathogen, causing a broad spectrum of diseases in healthy as well as in immunocompromised individuals (Ramanan et al. 2013).

The phenotypic identification of *Gordonia* genus species has been primarily based on biochemical tests (Verma et al. 2006). However, this methodology is time-consuming and frequently leads to misidentifications. Besides, new *Gordonia* species are continuously being described and biochemical tests commercially available for Gram-positive rods identification are not able to adequately classify all *Gordonia* at the species level. Therefore, gene sequencing has become the best technique to ensure a proper identification (Hsueh et al. 2014).

Matrix-assisted laser desorption ionization-time of flight (MALDI-TOF) mass spectrometry (MS) is an automated method recently implemented as a tool for bacterial identification in clinical microbiology laboratories. Based on poor laboratory results in the identification of *Gordonia* by MALDI-TOF MS some authors have suggested changing the cut-off points for *Gordonia* identification at the genus and species level, the proposed scores being ≥ 1.5 and ≥ 1.7 respectively (Rodríguez-Lozano et al. 2016; Barberis et al. 2014; Bizzini et al. 2010). This value system differs from the manufacturer’s recommendations which are score values ≥ 2.0 for identification at species level, between 2.0 and ≥ 1.7 at genus level and being a score of ≤ 1.7 considered as unreliable identification.

The aims of this work were to assess the implementation of MALDI-TOF MS for *Gordonia* species identification compared with 16S rRNA gene sequencing as the gold standard and to try to define discriminatory peaks for *Gordonia* species differentiation.

## Materials and methods

Between 1998 and 2015, 24 clinical isolates belonging to 21 different patients were identified as *Gordonia* at the Microbiology Department of Hospital Universitario Donostia, San Sebastian (Basque Country, Spain).Only one isolate per patient was considered for the study. Presumptive identification was established by the growth of characteristic salmon to orange colonies, coryneform Gram-positive rods appearance and non- or slightly-acid-fast bacilli. Biochemical identification was done using the API Coryne system (bioMérieux, France) which contained 20 miniaturized biochemical tests. Definitive species identification was established by sequencing a fragment of 1188 bp of the 16S rRNA gene using primers 5F (TGGAGAGTTTGATCCTGGCTAG) and 1193R (ACGTCATCCCCGCTTCCTT). A > 99% sequence identity with the sequences of *Gordonia* species available at GenBank using BLAST software (http://www.ncbi.nlm.nih.gov) was used as criteria for species identification.

Overall, *16 Gordonia sputi*, 4 *Gordonia otitidis and* 1 *Gordonia bronchialis* isolates were identified in sputum (n = 15), urine (n = 1) and blood (n = 5) samples from 21 patients (15 men, 6 women). In some patients the pathogenic role of *Gordonia* isolates could not be unequivocally ascertained, especially in those cases where the microorganism was isolated from sputum, given that *Gordonia* respiratory infections have been rarely described in literature (Brust et al. 2009) and patients suffered from other multiples illnesses.

Bacterial extracts for MALDI-TOF MS identification (Bruker, Daltonics, Germany) was carried out according to the manufacturer’s recommendations and results interpretation was done according to the Bruker score system and the proposed new scores. Measurements were carried out in triplicate and the average of the three results was used for analysis (Bizzini et al. 2010). Since *Gordonia* have been previously classified using biochemical tests as *Rhodococcus* (Tindall and Euzéby 2001; Lam et al. 2014), six *Rhodococcus equi* clinical isolates were also tested by MALDI-TOF to assess the possibility of misidentification with *Gordonia* species.

To determine discriminatory peaks for species identification, besides the spectra obtained for the 21 clinical isolates, the reference spectra of the following *Gordonia* reference strains available at the Bruker library were used: *Gordonia aichiensis*, *Gordonia alkanivorans*, *Gordonia australis*, *Gordonia bronchialis* (n = 2), *Gordonia rubripertincta* (n = 11), and *Gordonia sputi* (n = 4). All peak data were analyzed by the Pearson´s correlation matrix test performed with SPSS V24 software. Differences in the results obtained for *Gordonia* species identification using the two different score criteria were evaluated by the Fisher exact test using GraphPad Instat version 3.05 software (GraphPad Software Inc., La Jolla, CA, USA).

## Results

Using the 16S rRNA gene sequencing as standard and comparing the manufacturer’s and the new described score system for *Gordonia* identification, 10 isolates showing an unreliable identification (score < 1.700) were correctly identified at the genus level (Table 1) with the new system (p < 0.001) and 9 isolates correctly identified at genus level could be identified at the species level (p = 0.006). Two isolates were correctly identified by both methods at species level.

**Table 1:** *Gordonia* species identification by 16S rRNA gene sequencing and correlation with MALDI-TOF MS

Laboratory coding number	Molecular identification (16S rRNA gene)	GenBank accession number	Score	MALDI-TOF MS first species in the identification ranking list	Identification according to	
					Manufacturer’ scores	New scores
65206307	*G. bronchialis*	MH371019	1.899	*G. bronchialis*	Genus level	Species level
10999451	*G. sputi*	MH371338	1.864	*G. sputi*	Genus level	Species level
226573	*G. sputi*	MH368686	1.659	*G. sputi*	Unreliable identification	Genus level
228715	*G. sputi*	MH371337	1.633	*G. rubripertincta*	Unreliable identification	Genus level
234219	*G. sputi*	MH370805	2.05	*G. sputi*	Species level	Species level
239340	*G. sputi*	MH370806	1.742	*G. sputi*	Genus level	Species level
240919	*G. sputi*	MH370807	1.679	*G. sputi*	Unreliable identification	Genus level
241080	*G. sputi*	MH368687	1.703	*G. sputi*	Genus level	Species level
241362	*G. sputi*	MH370809	1.867	*G. sputi*	Genus level	Species level
241859	*G. sputi*	MH368688	2.12	*G. sputi*	Species level	Species level
241867	*G. sputi*	MH368689	1.549	*G. rubripertincta*	Unreliable identification	Genus level
241963	*G. sputi*	MH368690	1.72	*G. sputi*	Genus level	Species level
243347	*G. sputi*	MH370808	1.765	*G. sputi*	Genus level	Species level
243815	*G. sputi*	MH368691	1.768	*G. sputi*	Genus level	Species level
243837	*G. sputi*	MH368692	1.695	*G. sputi*	Unreliable identification	Genus level
245108	*G. sputi*	MH368693	1.64	*G. sputi*	Unreliable identification	Genus level
245419	*G. sputi*	MH368694	1.715	*G. sputi*	Genus level	Species level
240706	*G. otitidis*	MH371196	1.507	*G. rubripertincta*	Unreliable identification	Genus level
243669	*G. otitidis*	MH370799	1.674	*G. aichiensis*	Unreliable identification	Genus level
243814	*G. otitidis*	MH370800	1.638	*G. rubripertincta*	Unreliable identification	Genus level
245663	*G. otitidis*	MH370801	1.517	*G. rubripertincta*	Unreliable identification	Genus level

The four *G. otitidis* isolates were misidentified probably because of the absence of any *G. otitidis* reference isolate in the Bruker database.

None of the 6 *R. equi* isolates was misidentified as *Gordonia* although in only one case the MALDI-TOF MS identified the isolate as *R. equi* with a score value of 2.087. In the other five isolates the identification score was ≤ 1.2 not given a reliable identification despite the good peak profiles obtained.

To define discriminatory peaks for species identification, the spectra of 6 (15.4%) of the 39 different *Gordonia* species described up to date were employed. The Pearson correlation coefficient showed that most species were closely related with the exception of *G. rubripertincta* (r = 0.363) and *G. alkanivorans* (r = − 0.069). The spectra of *G. sputi* and *G. otitidis* clinical isolates were closely related (r = 0.946) and two discriminatory peaks at 3332.8 and 4117.5 m/z could be established. The presence of one of those peaks or both was enough to discriminate all *G. sputi* and *G. otitidis* isolates from *G. aichiensis*, *G. alkanivorans*, *G. australis*, *G. bronchialis*, and *G. rubripertincta* but not between them.

## Discussion

Of the 39 *Gordonia* species described to date only 10 have been identified as opportunistic pathogens in humans capable to cause a wide range of diseases ranging from primary cutaneous infections or lung infections to bacteremia or recurrent breast abscesses in healthy individuals as well as in immunocompromised patients (Sowani et al. 2017; Rodríguez-Lozano et al. 2016). *Gordonia* is difficult to identify using conventional biochemical tests as reflected in some reports (Tindall and Euzéby 2001; Lam et al. 2014) where all *Gordonia* isolates were misidentified as *Rhodococcus* species. To date, sequencing of the 16S rRNA gene has become the most reliable technique for *Gordonia* species identification (Bizzini et al. 2010). However, sequencing results are not generated instantly and are not available in all laboratories.

Recently, MALDI-TOF MS based system has emerged as new tool with the possibility to identify most of the microorganisms causing human infections (Lam et al. 2014). Different studies evaluated the precision of MALDI-TOF MS for *Gordonia* species identification with not very promising results (Hsueh et al. 2014; Rodríguez-Lozano et al. 2016; Barberis et al. 2014; Bizzini et al. 2010; Lai et al. 2010). In fact, based on previous studies (Rodríguez-Lozano et al. 2016; Barberis et al. 2014; Bartolomé-Álvarez et al. 2016), a lower score cut–off for an accurate identification at species and genus level was suggested. In our study, a lower score significantly increased the correct identification from 10 to 20 isolates at genus level and from 2 to 11 isolates at species level without affecting the specificity.

Our work, although with a limited number of different *Gordonia* species, confirmed that a lower cut-off score value was suitable for *Gordonia* genus and species identification. Also, the use of discriminatory peaks could aid in the differentiation of *G. sputi* and *G. otitidis* from other *Gordonia* species. Finally, it would be desirable, due to the increase in the description of new pathogenic *Gordonia* species, to increase the spectra of reference isolates in the MALDI-TOF databases to ensure a correct *Gordonia* species identification.
